# Basic Fibroblast Growth Factor for Treatment of Onychomadesis with Delayed Regrowth of the Nail

**DOI:** 10.1155/2013/214810

**Published:** 2013-06-23

**Authors:** Tomito Oji, Masaki Yazawa, Kazuo Kishi

**Affiliations:** Department of Plastic and Reconstructive Surgery, Keio University School of Medicine, 35 Shinanomachi, Shinjuku Ward, Tokyo 160-8582, Japan

## Abstract

Onychomadesis usually arises from an inflammation of the paronychium or as a result of blisters and hemorrhaging under a nail that has been struck or compressed. No documented interactions between basic fibroblast growth factor (bFGF) and onychomadesis have hitherto been reported. This case report describes a 25-year-old woman with onychomadesis following infection of the ingrown nail of her left thumb. After ten months of observation with no treatment showed no regrowth of her left thumbnail, the external use of bFGF and antibiotic ointment was started. One month later, nail regrowth was observed up to the halfway point of the nail bed, and after treatment for three months, the regrown nail reached the top of the nail bed. Both thumbnails now looked identical. This case suggests that external use of bFGF can promote nail regrowth in cases of onychomadesis with delayed regrowth of the nail.

## 1. Introduction

Onychomadesis usually arises from an inflammation of the paronychium or blisters and hemorrhage under the nail caused by a blow or compression. Systemic diseases, such as hand-foot-and-mouth disease, pemphigus vulgaris, and Stevens-Johnson syndrome, occasionally result in onychomadesis. Normally, simple observation or supportive treatment of inflammation will promote the regrowth of the fallen nail. However, in cases where nail regrowth does not occur, the condition tends to be refractory. Hitherto, there have been no effective treatments that promote the regrowth of fallen nails. 

In this paper, we report a notable case of onychomadesis after infection of an ingrown nail, in which the external use of basic fibroblast growth factor (bFGF; Trafermin; Fiblast Spray) and antibiotic ointment (Bacitracin-fradiomycin sulfate; Baramycin) led to normal nail regrowth.

## 2. Case Presentation

The case was a 25-year-old woman. She was a nursery teacher and routinely washed her hands many times daily. She had no previous medical history and was taking no medications or dietary supplements. Her left thumb had an ingrown nail, but she had not had it examined in a clinic, since it showed no troublesome symptoms. However, after her ingrown nail became infected, it presented with pain, swelling, and discharge of pus. Those symptoms continued for a month, after which the nail fell off the nail bed. She went to a clinic to have her left thumbnail examined at that time, but underwent observation rather than receiving treatment due to the expectation that it would spontaneously regenerate. No further infections flared up, but no regrowth of her left thumbnail was seen in four months.

**Figure 1:**
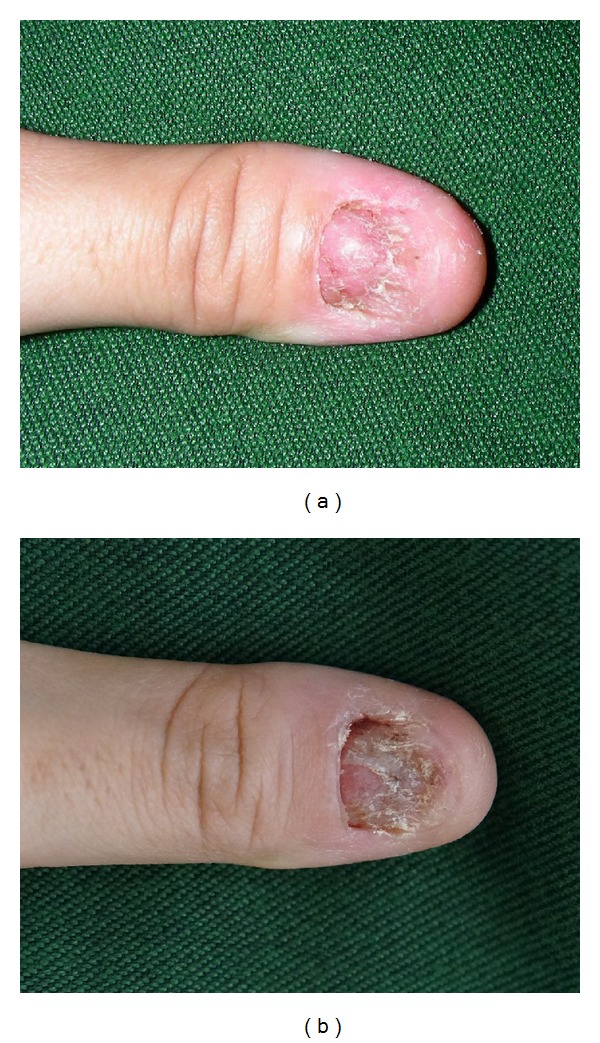
Left thumbnail five months after nail loss, at the first visit to our hospital (a) and 10 months after nail loss (b).

At her first visit to our hospital, five months after the nail loss, her left thumbnail was absent and the nail bed was entirely exposed (Figure 1(a)). We continued to observe it without treatment for the next six months; however, it showed no tendency toward regrowth. Her left thumbnail had thus shown no significant changes after 10 months of observation: the nail bed was still naked and showed no signs that it would regenerate (Figure 1(b)).

**Figure 2:**
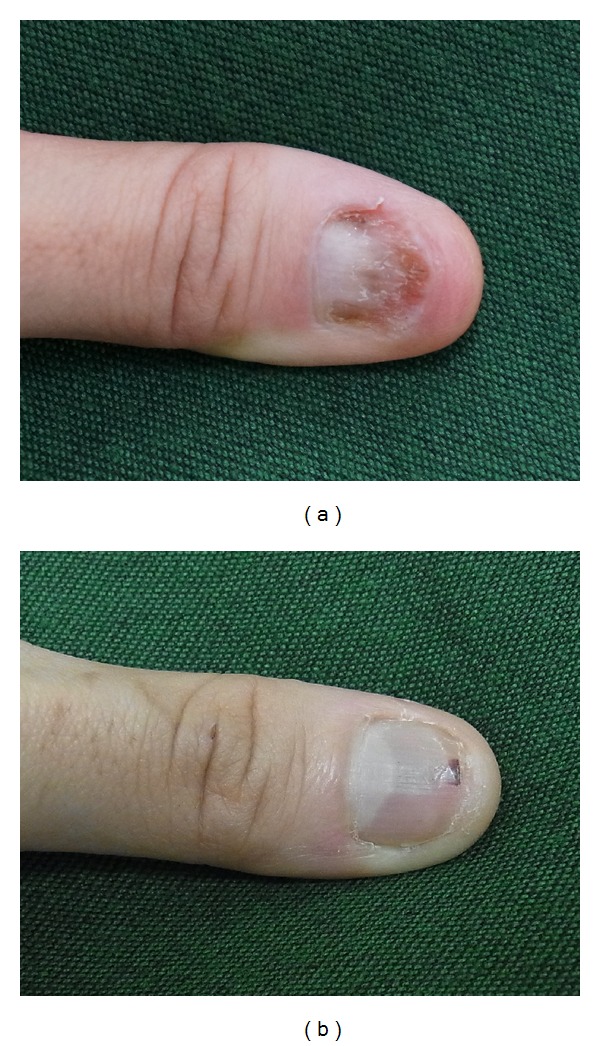
Left thumbnail one month after starting the external use of bFGF (a), and three months subsequently (b).

We therefore prescribed external application of bFGF recombinant and antibiotic ointment: five applications of the bFGF spray to both the nail bed and the nail matrix once a day, and application of the ointment on the same site twice daily to ensure that it did not dry out. One month after beginning the treatment, nail regrowth was observed up to the halfway point of the nail bed (Figure 2(a)). After three months of the same treatment, the regrown nail reached the top of the nail bed, and there was no visible difference between her two thumbnails (Figure 2(b)).

## 3. Discussion

Normal nails are formed by nail matrix keratinocytes that undergo cell division below the nail roots. If these nail matrix keratinocytes are somehow damaged, the nails may fall out of their nail beds. In the case of our patient, we assumed that an inflammation had occurred in the nail matrix keratinocytes following the infection of the ingrown nail, leading to loss of the nail. 

Basic fibroblast growth factor (bFGF) is a protein that was isolated from bovine pituitary glands in 1974 [1]. It promotes the cell division of fibroblasts. Recent studies have revealed that bFGF affects various kinds of cells in many organs during cell division, including vascular endothelial cells, vascular smooth myocytes, and corneal endothelial cells [2]. In wound healing, bFGF acts chiefly on the fibroblasts, vascular endothelial cells, and epidermal keratinocytes to promote healing [3, 4]. Recent studies have revealed further functions of bFGF: a promotive effect on nerve regeneration [5] and a depressive effect on scar contracture [6].

In this case of onychomadesis, it appears that treatment with bFGF activated nail regeneration. It might also promote the proliferation of nail matrix keratinocytes, since it is known to act on epidermal keratinocytes. To our knowledge, ours is the first study to report that the external use of bFGF can promote nail regrowth.

Our study shows that external use of bFGF appears to stimulate nail regrowth in the case of onychomadesis after infection of an ingrown nail. To more fully assess the external effect of bFGF on nail regrowth, we believe there is a need for further case reports on the clinical use of bFGF to treat delayed regrowth of nails caused by onychomadesis.

## 4. Conclusion

We report here an interesting case of onychomadesis, in which external use of bFGF motivated nail regrowth, in spite of the nail showing no tendency to regrow after 10 months of observation. This case also suggests that bFGF, when applied externally, can promote nail regrowth in cases with delayed nail regeneration after onychoptosis.
